# Sex-related interannual plasticity in wing morphological design in *Heliconius charithonia* enhances flight metabolic performance

**DOI:** 10.1371/journal.pone.0239620

**Published:** 2020-10-30

**Authors:** Velia I. Ramos-Pérez, Ignacio Castellanos, Virginia A. Robinson-Fuentes, Rogelio Macías-Ordóñez, Luis Mendoza-Cuenca

**Affiliations:** 1 Laboratorio de Ecología de la Conducta, Facultad de Biología, Universidad Michoacana de San Nicolás de Hidalgo, Morelia, Michoacán, México; 2 Laboratorio Nacional de Análisis y Síntesis Ecológica, ENES, UNAM, Morelia, México; 3 Centro de Investigaciones Biológicas, Universidad Autónoma del Estado de Hidalgo, Mineral de la Reforma, Hidalgo, México; 4 Facultad de Ciencias Médicas y Biológicas "Dr. Ignacio Chávez", Universidad Michoacana de San Nicolás de Hidalgo, Morelia, Michoacán, México; 5 Red de Biología Evolutiva, Instituto de Ecología, A.C., Xalapa, Veracruz, México; USDA Agricultural Research Service, UNITED STATES

## Abstract

Flight morphological variations and its consequences on animal performance are common in winged insects. In the butterfly *Heliconius charithonia*, sex-related differences in the wing morphological design have been described resulting in differences in foraging behavior, daily flight distances and flight aerodynamics. It has been suggested that these differences should be reflected in the metabolic capacities and energetic budgets associated with flight in both sexes. In this study, we analyzed the relationship between wing morphological variation and metabolic performance, flight aerodynamics and energetic reserves in females and males of *Heliconius charithonia* over two years. The results confirm the presence of wing shape sexual dimorphism, but also show an unexpected sex-related annual variation in wing shape, mirrored in the metabolic condition (resting metabolic rate) of individuals. However, contrary to expectation, intersexual variations in wing shape are not related to differences between the sexes in terms of flight aerodynamics, flight metabolic rates, or energetic reserves (carbohydrates, lipids and proteins). Our results indicate a considerable plasticity in *H*. *charithonia* wing shape, which we suggest is determined by a trade-off between environmental pressures and reproductive restriction of each sex, maintaining an optimum flight design. Finally, similarities in metabolic rates between young and older males and females in both years may be a consequence of the ability of *Heliconius* species to feed on pollen.

## Introduction

Flight is a costly activity for insects, for which the condition and aerobic capacity of the individual, as well as the availability of fuel, are important factors in the locomotor effort involved for take-off, sustained flight, evasive maneuvers and landing [1–3]. Insect flight muscles require considerable metabolic and energetic expenditure [2, 4]; therefore, performance depends on the capacity of each individual for oxygen conductance to flight muscles and storage of flight energy sources (e.g. carbohydrates and lipids) [4]. These fuel sources are obtained through feeding and are degraded and synthesized by different processes at cellular level in order to produce the energy for flight. In this sense, the almost immediate capacity for flight from a resting state in the insects is limited by the availability of an adequate oxygen supply and the presence of energetic reserves in the flight muscles, and produces metabolic rates that are up to 100 times greater than those at rest [1–3, 5]. For this reason, an important trade-off exists in flying insects between the energy spent in flight and that assigned to somatic maintenance and reproduction.

In the context of this energetic trade-off, insect flight is highly efficient, due mainly to the flexibility of the anatomic structures necessary for flight, such as the wing membrane [2, 3] and to particular kinematics of their flight that confer great maneuverability [6]. In butterflies, it is necessary to generate enough force to support the body mass and maneuver through the constant movement of the wings and against the resistance of the forces imposed by the surrounding air as well as those of the inertia associated with continuous acceleration and deceleration of the corporal mass [5, 7]. For this reason, studies of flight aerodynamics focus on the evaluation of parameters such as the capacity of the individual to carry its own body mass and produce sufficient force to enable flight (i.e. wing loading) [2, 8–10], the proportion of flight muscle as an estimator of the musculature necessary to support the mass of the individual as well as its capacity for acceleration [8, 11] and descriptors of the wing shape such as the aspect ratio [2, 11, 12].

The energy required by butterfly flight muscles is primarily obtained from carbohydrates and lipids present in food and long-term energetic reserves, which are converted to energy through cellular metabolization [1, 3–5]. For this reason, energetic reserves are considered a suitable estimator for the energetic condition, muscle investment and flight power of an individual (i.e. the proportion of lipids present in the thoracic muscles) [13]. Furthermore, determining the metabolic expenditure of an individual through measurement of metabolic rates provides an estimate of the rate at which an individual uses the energy available (i.e. energetic reserves) in a state of complete rest (i.e. resting metabolic rate) as an indicator of corporal condition, or during some activity (e.g. flight metabolic rate), in order to estimate flight cost. This is reflected during respiration in the consumption of oxygen or production of carbon dioxide [1–3, 5].

Several studies have focused on evaluation of metabolic and energetic performance in insects [14–19] and their relationship with factors such as wing shape, age and fecundity [20–24]. A few studies have even reported effects related to senescence [25]. Some works have also addressed the relationships among morphological variation, metabolic expenditure during flight and the use of the energetic reserves of wild individuals [13, 24, 26–28]. The study of the morphology of flight is of critical importance in flying insects such as butterflies, which involve flight in almost all of their activities (i.e. feeding, searching for mates, dispersion, oviposition) and in which a wide intra- and interspecific variation in wing morphologies associated with different flight behaviors exist [2, 26, 29]. In this sense, recent evidence in butterflies shows that the environmental conditions experienced by the larvae (e.g. temperature, availability of food, landscape structure) act to modify adult wing shape, allometry among traits and the aerodynamic and metabolic performance of individuals and thus their behavior [30, 31].

Butterflies of the genus *Heliconius* present a wide diversity of adaptive wing morphologies as a result of different pressures of natural and sexual selection. This, added to their considerable longevity (i.e. 3 to 6 months) and elevated energetic budgets as a result of presenting the rare behavior among butterflies of feeding on both pollen and nectar [32–36], makes them a model group for study of the relationships among wing morphology, morphological flight performance and its effect on metabolism. In this context, in all *Heliconius* species in which reproductive behavior has been studied, a close relationship has been observed between the reproductive strategies of the males and variations in wing morphology [37, 38]. For *Heliconius charithonia* in particular, a long-term study described two reproductive phenotypes in the males: some perform pupal mating and may present elongated and wide wings that improve their capacity to physically exclude other males in competition for mating with females in the pupal stage, while other males with short slender wings show greater maneuverability and practice a reproductive strategy of pursuit of flying adult females [11, 39]. Conversely, females present wing lengths similar to the pupal males, but with greater wing loading, allowing them to carry the additional mass of the ovules present in the ovaries [11].

In this species, the essential amino acids available in pollen are transferred to the production of eggs and spermatophores, and increase the longevity and fertility of individuals [40–42]. However this represents an important trade-off since pollen feeding is a time demanding process [32–34], in which *H*. *charithonia* females spend several hours a day, and the time increases with the age of the females. Males however spent most of their time searching for female pupae and have low foraging rates despite engaging in costly reproductive flights (e.g. hovering) and daily long distances travel.

It has been suggested that these variations should lead to large intersexual differences in the metabolic and energetic costs of flight in *H*. *charithonia* and be reflected in asymmetric levels of energetic reserves [11]. Therefore, in this study we evaluated the patterns of inter and intrasexual dimorphism in wing morphology in two consecutive years, as well as their relationship with flight metabolic cost (i.e. energetic cost) and energetic condition in *H*. *charithonia*. Specifically we predicted lower metabolic rates associated with lower quantity or quality of energetic reserves in males, while females would present higher metabolic rates and high quantities of energetic reserves allowing them to cover the energetic cost of producing and carrying eggs.

## Materials and methods

The study was conducted in a population of *H*. *charithonia* located in a remnant of tropical montane cloud forest encompassing the Santuario del Bosque de Niebla and the Francisco Javier Clavijero Botanical Garden, in the city of Xalapa, Veracruz, Mexico (19° 30’N, 96° 56’W; mean elevation 1400 masl), over two consecutive years in the season of peak abundance of this species [11]: October 2016 and September 2017. Age can modify the metabolism and energetic reserves of individuals. For this reason we tried to limit the age range of individuals in the study and collected exclusively “young individuals” of an age range 5–15 days, which show complete and totally hardened wings, little or no loss of scales and bright yellow and black coloration (L. Mendoza-Cuenca, unpublished data). In each season, the study site was visited daily for 30 days between 09:00 and 14:00 h CDT (GMT-5). During the first 20 days, all of the young individuals observed flying in the area were collected. All individuals were collected during or immediately after feeding on *Hamelia patens* or *Dahlia coccinea* flowers, which are the most common pollen resources available in the study area [11]. These specimens were marked with consecutive numbers (marks on the anterior left wing of the females and right wing of the males), using a permanent marker pen of white ink, with ultrafine tip, sexed, photographed and the day of marking noted (assigned as the day of age 1 of each individual). They were then released for at least 10 days, during that period most of them were seen flying and feeding daily in the area, and then captured to measure metabolic rate and then sacrificed to weigh and estimate energetic reserves.

A total of 129 individuals were marked and photographed in the two years: October 2016 (18 females and 35 males) and September 2017 (37 females and 39 males).

### Permits obtained for work

Both study sites “Santuario del Bosque de Niebla” and “Francisco Javier Clavijero Botanical Garden” are under the protection of the Instituto de Ecología, A.C. and the permits for access and collection of specimens were granted by its technical secretary M. Sc. Orlik Gómez García. This study was carried out in strict accordance with the recommendations of the “Regulation of the Institutional Ethics Committee” of the Universidad Michoacana de San Nicolás de Hidalgo.

### Wing morphology

**Fig 1:**
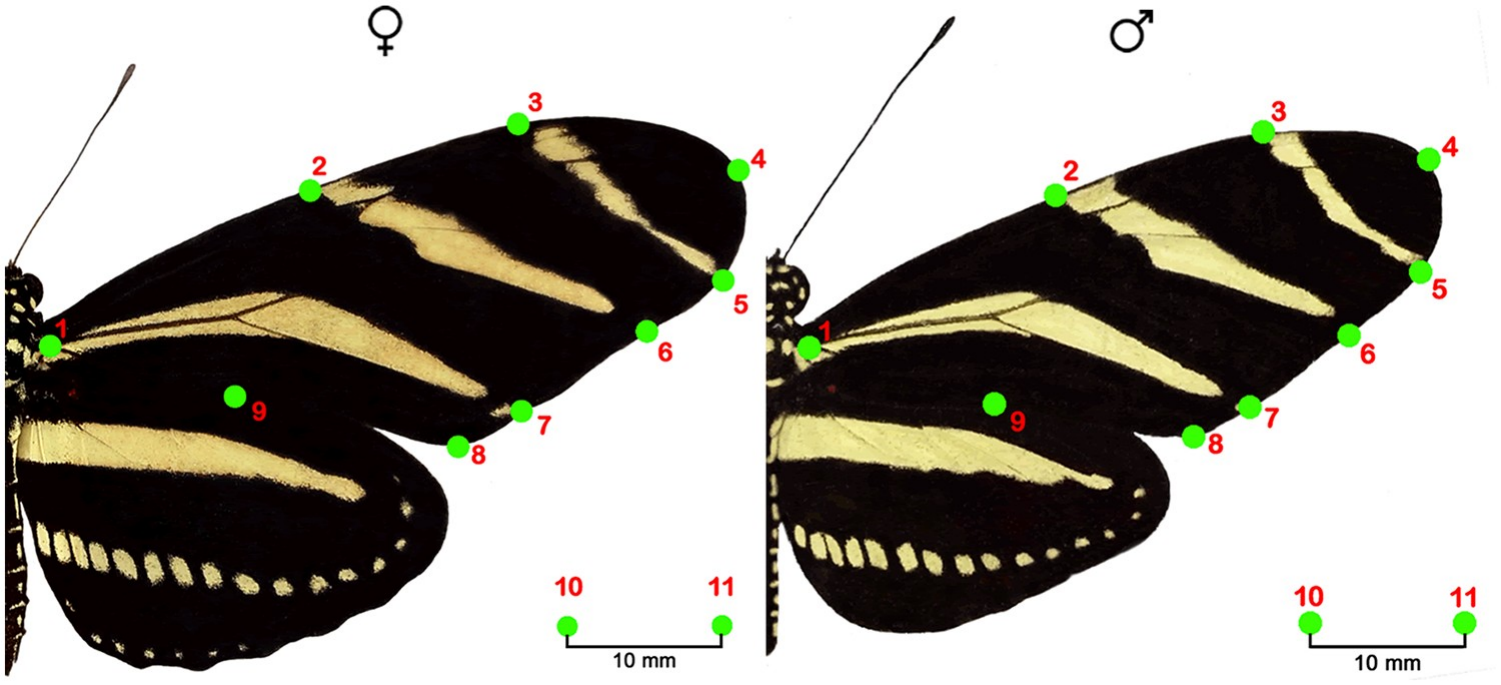
Position of the landmarks on the right forewing of females and males of *H*. *charithonia*.

Photographs were taken of each specimen with a 13-megapixel digital camera (MotoG3) on a fixed base with a millimetric scale in order to maintain the same focal distance for each individual. The individuals were carefully immobilized and posed with the two wings extended in flight position. The photographs were analyzed using techniques of geometric morphometry in order to compare wing shape between males and females. Nine anatomical markers (i.e. landmarks) were placed on the outline of the right forewing and on the intersection with the lines of yellow color (Fig 1), which have been proposed as homologous landmarks on all individuals of both sexes [3] using the program TPSdig [43]. Two points were also placed on the millimetric scale as size reference. A Procrustes superimposition was conducted in order to generate the coordinates that were used as variables of shape, using the program CoordGen8 [44]. A canonical analysis of variables was performed to compare the shape of the male and female wings in both sampling periods, using the program CVAGen8 [44]. The estimators were analyzed separately, between sexes and years, in order to control for the effect of the year in which the collections were made. Furthermore, to assess whether wing shape exhibits seasonal plasticity, we compare wing shape between youngest and oldest individuals (see details of age determination in resting metabolic rate section) of each sex and year by performing Bootstraped Procrustes F-test (1000 bootstraps) using the program TwoGroup8 [44].

### Flight aerodynamics

The image processor ImageJ 1.50i was used to determine wing length (base to tip of right forewing), total body length (tip of head to tip of abdomen), and wing area as estimators of body size. In addition, total dry mass was determined in each individual, as well as the mass of the thorax and abdomen (both separately) with no legs or wings, using an analytical balance (Sartorius CP124S) of precision 0.01 g. The total dry body mass and wing area values were used to calculate the wing loading of each individual as an estimator of the carrying capacity of the wing membranes [2, 8–10]:

$$Wing\;loading=\frac{9.81*total\;dry\;mass}{wing\;area}$$

The proportion of flight muscles (thoracic weight) was used as an estimator of the quantity of muscles necessary to support the mass and to accelerate [8, 11]:

$$Proportion\;of\;flight\;muscles=\frac{Thoracic\;weight}{body\;weight}$$

The aspect ratio was used to describe the wing shape [2, 11]:

$$Aspect\;ratio=\frac{4{\left(wing\;length\right)}^{2}}{wing\;area}$$

Differences in aerodynamic flight capabilities were analyzed using two-way ANOVAs with size and aerodynamic measures as dependent variables, and year and sex as independent factors in the program JMP 8.0. The normality and homogeneity of variances of the data were evaluated, and all data fulfilled both assumptions.

### Metabolic condition

The metabolic condition of each individual was evaluated using a continuous flow respirometer (Q-Box RP1LP Low Range Respirometry Package, Qubit Systems Inc., Kingston ON, Canada), adjusted to a constant airflow of 300 ml/min, and a cylindrical G115 Flow-through chamber (3.8 x 20 cm; 226.82 ml). The air that entered the chamber was depurated of CO_2_ with “soda lime” (CaHNaO_2_) in order to ensure that all CO_2_ detected was that produced by the respiration of the individuals under study [45]. Two condition measurements were taken for each individual: the first to evaluate the resting metabolic rate of the individual, and the second to assess the effort represented by sustained flight in individuals of *H*. *charithonia* of both sexes.

#### Resting metabolic rate (RMR)

Starting on the 21st day of each sampling period, all previously marked individuals still flying in the area were collected, and their age was estimated (i.e. number of days elapsed since marking). Two to five individuals were collected daily and placed in a polyester cylindrical mesh cage, 100 cm in height and 38 cm in diameter, in a dark room at ambient temperature to induce a resting state and reduce stress. The specimens were placed randomly and individually within the chamber of the respirometer, covered with a dark cloth in order to reduce the stimuli that could influence the activity of the individuals. Initially, tests with readings of 20 minutes were conducted. However, since the resting metabolic rate is constant from the first 2 minutes onwards, and in order to reduce any collateral effect of the measurement (e.g. stress, dehydration), the measurement time was set to seven minutes, and we used the average of 1 min (i.e. fifth minute) of stable volume of CO_2_ production (ml/h), the period of the lowest activity and the greatest stability of CO_2_ emission as an estimator of the resting metabolism [24]. The experiment was conducted during the time of day of low or null activity of the species in the study zone (18:00 to 20:00 h). The individuals were fed with a 10% sugar solution and maintained in the same conditions until the following measurement. Given that it was not possible to accurately establish the age of the individuals on initial capture, and that this factor could modify the RMR [13, 23, 25], we considered the age of the individuals as the time between the day of marking and the measurement of the RMR and used linear regressions per sex and sampling period to evaluate whether there was a relationship between age and RMR.

#### Post-flight metabolic rate (PFMR)

Since it was not possible to measure actual flight metabolic rate in *H*. *charithonia* we assessed post-flight metabolic rate as estimator of the metabolic cost of flight activity after a brief flight [46, 47]. The day following measurement of the RMR, each individual was selected randomly and placed in a new polyester cylindrical mesh cage (70 x 120 cm), in which the butterfly was forced to fly continuously for a period of 90 seconds preventing them from landing by blowing short bursts of air with a manual pump if necessary. This time period was chosen considering that individuals of both sexes of *H*. *charithonia* in the wild make short foraging flights (ca. 100 m in distance) (L. Mendoza-Cuenca, pers. obs.) with speeds of 2–2.7 m s^-1^ in natural environments [48] and of 1.4 m s^-1^ in artificial environments [49]. During the experimental trial of 90 seconds, an individual of *H*. *charithonia* could cover approximately 120 m. We therefore considered that 90 seconds would represent a typical flight for individuals of this species and would thus avoid an unwanted effect of exhaustion being reflected in the measurement of the PFMR [23]. Each individual was then immediately taken from the cage and placed in the chamber of the respirometer for seven minutes to measure the PFMR. Transfer time of each butterfly from the flight cage to the respirometer chamber and the start of PFMR measurement was never greater than 10 seconds because the respirometer was less than a meter apart from the flight cage and while one person captured and transferred the butterfly to the chamber, another opened and closed the chamber just long enough to introduce the individual). The reading of CO_2_ production was taken from the second minute in order to avoid an erroneous reading in the first minute, since opening the respirometer chamber to introduce the individual allows the entry of CO_2_ from the respiration of the observer. As an estimator of the PFMR, the integral of the effort was used (i.e. total CO_2_ production from the second minute of post-activity up until seven minutes were completed within the chamber of the respirometer [50], as well as the PFMR maximum (PFMRmax; average of the second minute of CO_2_ production) [46].

The flight effort (PFMR) was recorded during the time of activity of the individuals (10:00 to 14:00 hrs). All of the metabolic data were obtained using the software Logger Pro3.8.4 (Vernier Software & Technology) and converted using the equations suggested by Lighton [45]. At the end of the flight effort experiment, the individuals were sacrificed in order to quantify their content of lipids, proteins and carbohydrates as a measure of the energetic budget stored by the individuals.

### Energetic reserves

The thorax and abdomen of each individual were crushed separately; 5 mg of muscle and exoskeleton were taken from the thorax and 5 mg from the abdomen. The samples were prepared independently. The method proposed by Mokrasch [51] were followed for the determination of carbohydrates, the method of Goldsworthy *et al*. [52] for the determination of lipids, and the method of Le Bras and Echaubard [53], and the Coomassie blue method [54] for the determination of proteins. Quantification of carbohydrates, lipids and proteins was conducted in a spectrophotometer (Spectronic 20 genesys) at wavelengths of 625, 530 and 595 nm, respectively. Trehalose, cholesterol and bovine albumin were used as standards and the results were expressed as μg of the compound per milligram of tissue.

In order to evaluate the relationship between RMR, PFMR, flight aerodynamic variables and energetic reserves, we first generated flight performance estimators and budget estimators using two principal components analysis. In the first PCA we used the aerodynamic variables wing length, body length, dry abdominal mass, dry thoracic mass, total dry mass, wing loading, proportion of flight muscles and aspect ratio. In the second PCA we used the energetic variables: carbohydrates, lipids and proteins in both thorax and abdomen, and the proportion of lipids in the thoracic muscles. The program JMP 8.0 (SAS, Institute Inc) was used to perform these analyses.

The first three principal components of the flight performance estimator and the first three of the energy budget estimator were used as independent variables to analyze the effect of flight aerodynamics and energetic reserves both on RMR and FMR, using two generalized linear models (GLM_RMR_ and GLM_PFMR_) with a gamma error distribution and a log link function. The models considered year, sex and principal components as fixed factors, and volume of CO_2_ (**ml/h**) as a response variable.

The proportion of lipids present in the thoracic muscles of all individuals (i.e. dry thoracic mass/dry body mass remaining following lipid extraction) was calculated as an estimator of the investment in musculature and power of flight [13]. A generalized linear model with a quasibinomial error distribution was performed, with year and sex as independent variables and proportion of lipids as the response variable. The R statistical package version 3.3.1 [55] was used for these analyses.

### Relationship between wing shape and metabolic and energetic expenditure

Using the Procrustes coordinates as a matrix of shape, a multiple partial least squares (PLS) regression was conducted to analyze the relationship between the wing shape of males and females (dependent variables) and the aerodynamic (dry body mass, wing length, wing loading, proportion of flight muscle and aspect ratio), metabolic (resting metabolic rate, post flight metabolic rate and PFMR maximum) and energetic (carbohydrates, lipids, proteins and the proportion of lipids present in the thoracic muscles) variables as independent variables. PLS analyses were performed using the programs PLSMaker8 [44] and R 3.3.1 [55], with the library Geomorph [56].

## Results

### Wing morphology

**Fig 2:**
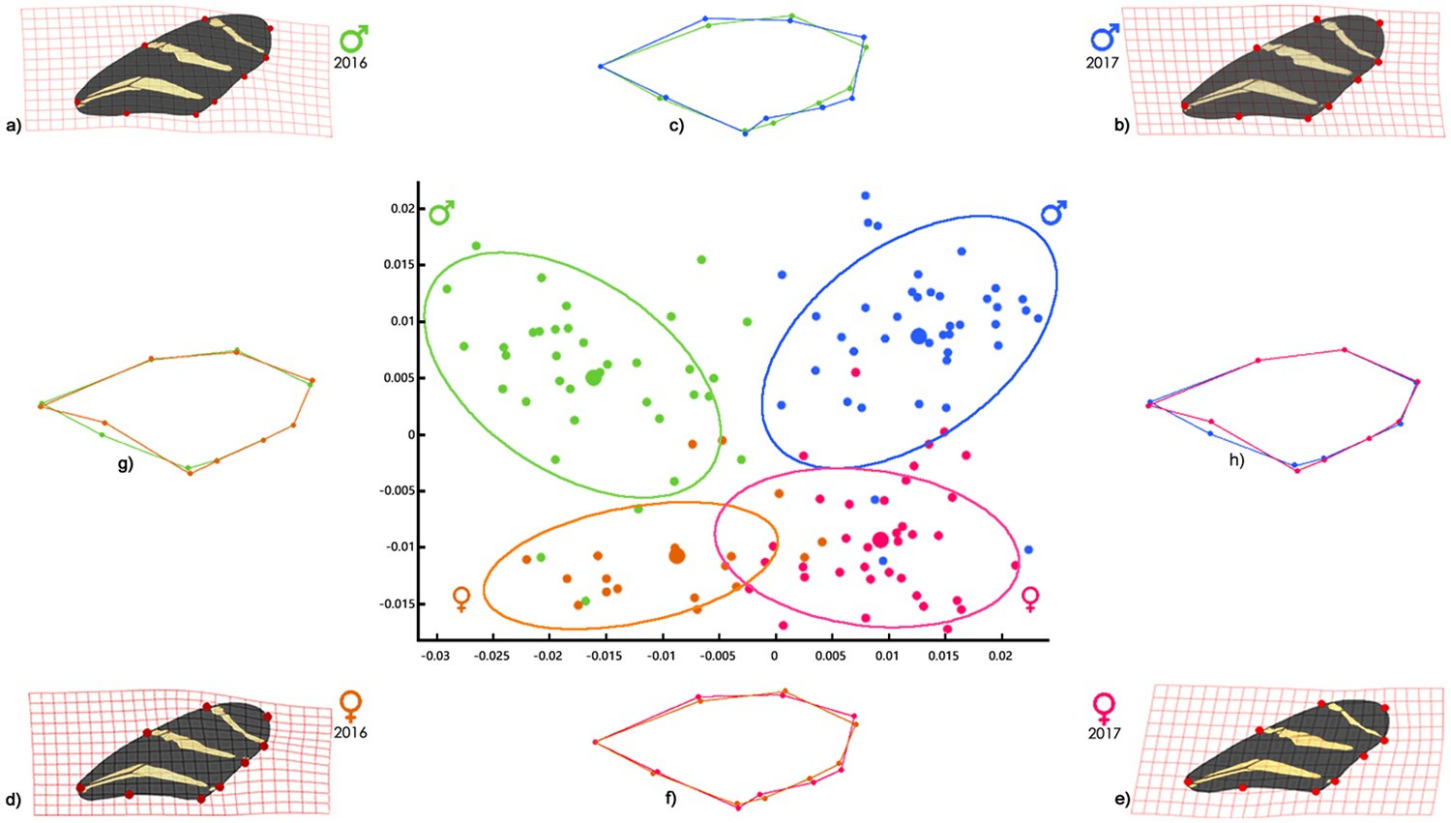
Canonical variate analysis of all of the individuals of *H*. *charithonia* collected in 2016 and 2017. Deformation grids of: a) males of 2016, b) males of 2017, d) females of 2016 and e) females of 2017. Thin plate spline comparisons: c) males of 2016 vs. males of 2017, f) females of 2016 vs. females of 2017, g) females of 2016 vs. males of 2016 and h) females of 2017 vs. males of 2017.

The Canonical Variates Analysis conducted to determine wing shape in individuals of *H*. *charithonia* showed no significant differences in shape between females of both collections (i.e. 2016 and 2017) (Fig 2), but did show significant differences between females and males in each year of sampling (Fig 2) and between the males of both sampling years (Axis 1: Lambda = 0.06, chisq = 338.87, d.f. = 42, *p*<0.001; Axis 2: Lambda = 0.29, chisq = 148.48, d.f. = 26, *p*<0.001; Fig 2). Thin plate spline analysis showed that wings of the males of 2016 were slenderer, than the wider and longer wings of the males of 2017 (Fig 2a–2c). In the case of the wing shape in the females, no significant differences were observed between sampling years (Fig 2d–2f). On comparing the wing shape of the females of each sampling year, relative to that of the males of the same year, the wing shape in the females was observed to be thinner close to the base of the wing, compared to that of males (Fig 2g and 2h).

Bootstrapped Procrustes F-tests to assess seasonal wing shape, showed no significant differences in any comparisons between young and old individuals (Females 2016, F-Score = 0.74, p = 0.59; Males 2016, F-Score = 1.25, p = 0.21; Females 2017, F-Score = 1.46, p = 0.22; Males 2017, F-Score = 1.69, p = 0.12).

### Flight aerodynamics and energy reserves

**Fig 3:**
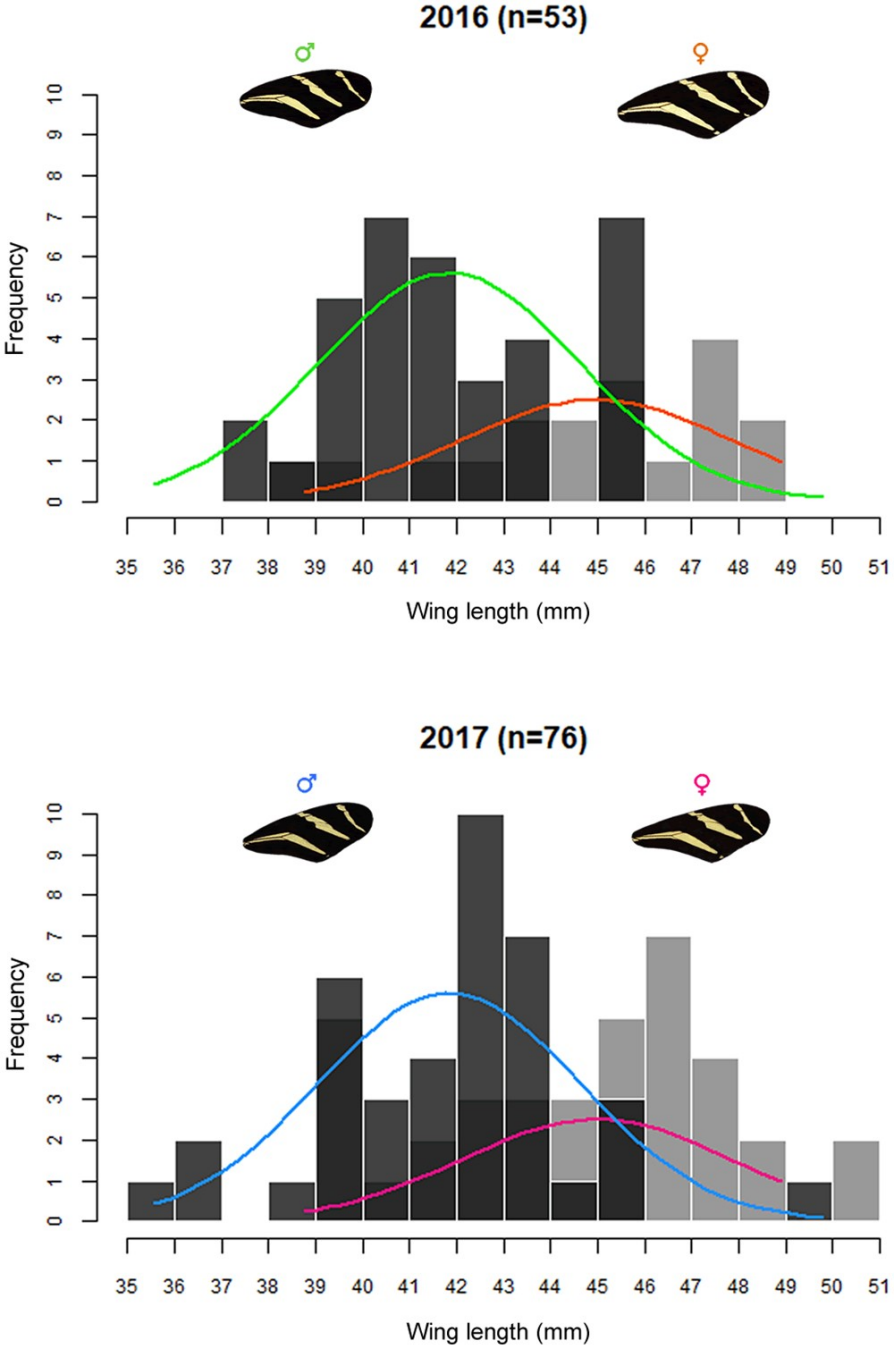
Wing length in individuals of *H*. *charithonia*. Seasons 2016 (female distribution represented by orange line and male distribution represented by green line) and 2017 (female distribution represented by pink line and male distribution represented by blue line).

Size measurements (i.e. wing and body length) were obtained for all individuals photographed (N = 129, Fig 3), while the aerodynamic variables were measured only in those individuals on which metabolic rate was measured (N = 51, S1 Table).

The two-way ANOVA showed significant differences in wing size between sexes but not between years (S1 Table), female wings were larger than male wings in both years (S1 Table; Tukey-Kramer post hoc test 〈 = 0.05). While for body size, the only significant differences were between females of 2017 and males of 2016 (S1 Table). Regarding flight aerodynamics analysis, the two-way ANOVAs revealed no effect of either year or sex on any of the estimated variables (S1 Table).

The Principal Components Analyses conducted considered eight aerodynamic variables in one analysis, and seven energetic variables in other (21 females and 30 males, S2 Table). In the analysis for aerodynamic variables, three principal components were obtained that together explained 85.59% of the total variance (Table 1). In the second Principal Components Analysis, for the energetic variables, three principal components were found that together explained 80.66% of the total variance (Table 2).

**Table 1:** Results of the PCA for aerodynamic variables in *H*. *charithonia* showing the contribution of the aerodynamic variables in the first three principal components.

	Principal Component		
Aerodynamic Variables	PC1 60.45%	PC2 13.83%	PC3 11.31%
Wing length	0.378	-0.043	-0.488
Body length	0.345	0.048	-0.551
Dry abdominal mass	0.383	-0.422	0.049
Dry thoracic mass	0.435	0.192	0.163
Total mass	0.444	-0.122	0.145
Wing loading	0.343	-0.159	0.616
Proportion of flight muscles	0.171	0.841	0.147
Aspect ratio	-0.236	-0.177	0.069

**Table 2:** Results of the PCA for energetic variables in *H*. *charithonia* showing the contribution of the energetic variables in the first three principal components.

	Principal Component		
Energetic Variables	PC1 52.57%	PC2 17.42%	PC3 10.67%
Carbohydrates in thorax	0.42	-0.343	0.182
Carbohydrates in abdomen	0.407	-0.382	0.07
Lipids in thorax	0.274	0.434	-0.736
Lipids in abdomen	0.437	0.046	-0.179
Proteins in thorax	0.434	0.012	0.008
Proteins in abdomen	0.424	0.102	0.201
Proportion of lipids in thoracic muscles	0.145	0.711	0.588

### Metabolic condition

The resting (RMR) and post-flight (PFMR) metabolism rate values were measured as produced CO_2_ volume (**ml/h**) in all marked individuals recaptured at least 10 days after marking (i.e. 21 females and 30 males; S2 Table). The individuals used to measure RMR were therefore all within an age range of 15 to 45 days (see methodology section). For this reason, simple linear regressions were performed for each year in order to determine whether there was an effect of age on RMR. The linear regressions showed no relationship between age and RMR in the females of 2016 (r^2^ = 0.03, F = 0.20, p = 0.67) or 2017 (r^2^ = 0.11, F = 1.60, p = 0.23), nor in the males of 2016 (r^2^ = 0.03, F = 0.33, p = 0.58) or 2017 (r^2^ = 0.03, F = 0.49, p = 0.49).

**Fig 4:**
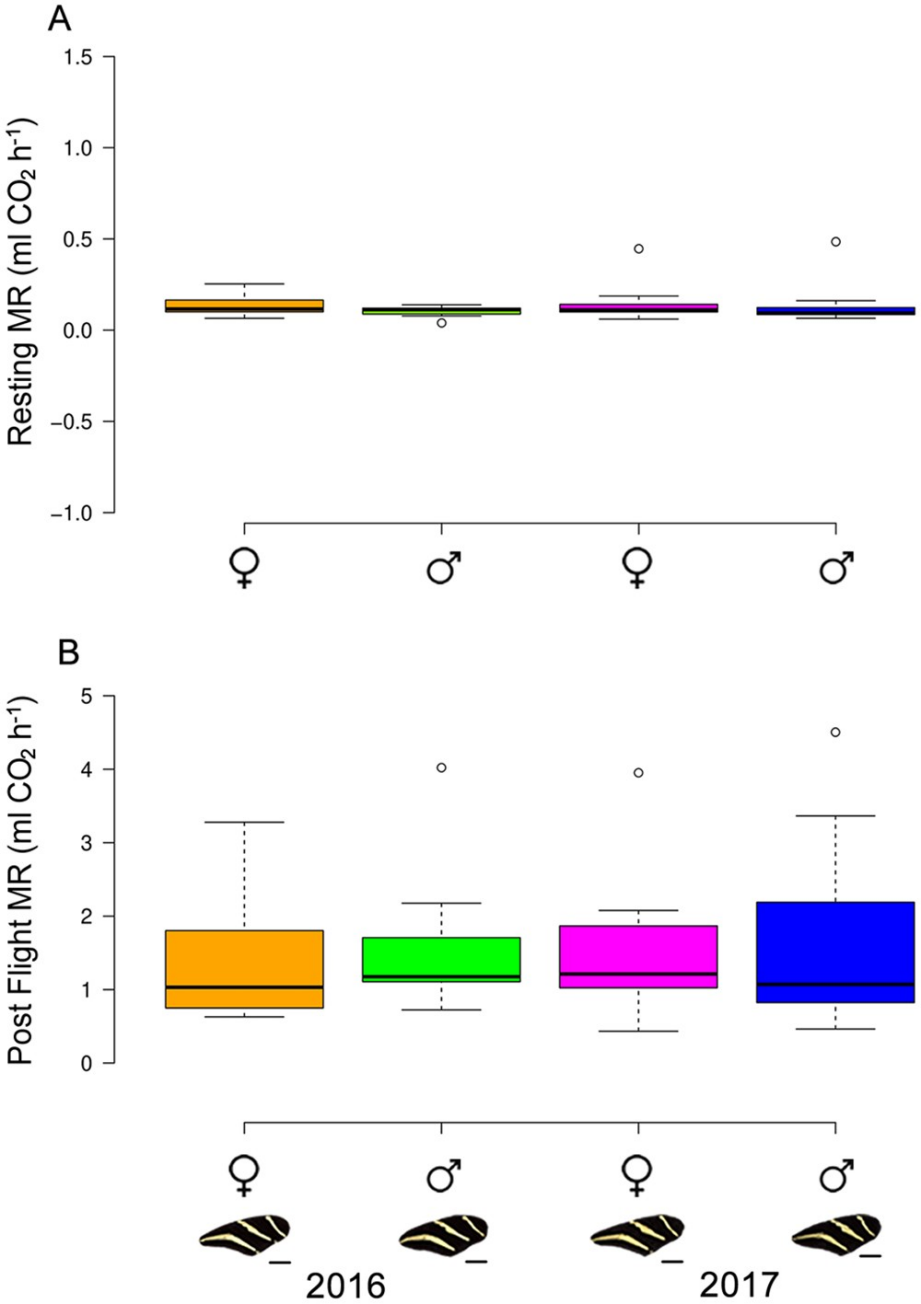
Comparison of metabolic rates in *H*. *charithonia* between sexes and years. A) Resting metabolic rate, B) Post-flight metabolic rate. Plots contains raw metabolic rate values.

The results of the GLM_RMR_ showed significant effects only for the first principal component (i.e. Total mass, thoracic mass, aspect ratio) in the CO_2_ volume production for resting individuals (Table 3, Fig 4). The GLM_PFMR_ also showed significant effects only for the first principal component in the volume of CO_2_ produced after a flight test (Table 3, Fig 4).

**Table 3:** Generalized linear models effects on RMR and PFMR.

	RMR			PFMR		
Parameters	Df	Residual Deviance	Pr(<X^2^)	Df	Residual Deviance	Pr(<X^2^)
**Year**	1	0.14150	0.3840	1	0.0000	0.9998
**Sex**	1	0.43144	0.1284	1	0.0835	0.6138
**AC1**	1	2.28585	**0.0004**	1	4.7000	**0.0001**
**AC2**	1	0.00081	0.9473	1	0.1179	0.5487
**AC3**	1	0.37394	0.1570	1	0.0816	0.6179
**EC1**	1	0.24377	0.2532	1	0.0016	09436
**EC2**	1	0.45451	0.1199	1	0.1836	0.4542
**EC3**	1	0.35990	0.1650	1	0.0020	0.9379

The complete models are: GLM_RMR_: glm((RMR) ~ (Year+Sex+A.C1+A.C2+E.C1+E.C2+E.C3), family = gamma, link = “log”) and GLM_FMR_: glm((FMR) ~ (Year+Sex+A.C1+A.C2+E.C1+E.C2+E.C3), family = gamma, link = “log”). Significant factors in bold.

*A.C1, A.C2 and A.C3 (first two principal components of the flight performance estimator).

E.C1, E.C2 and E.C3 (first three principal components of the energy budget estimator).

The GLM of the proportion of lipids present in the thoracic muscles revealed no significant differences neither for year (Residual deviance = 0.149, d.f. = 1, p = 0.789), sex (Residual deviance = 0.0006, d.f. = 1, p = 0.788) or the year by sex statistical interaction (Residual deviance = 0.008, d.f. = 1, p = 0.779).

### Relationship between wing shape, metabolic and energetic expenditure

**Fig 5:**
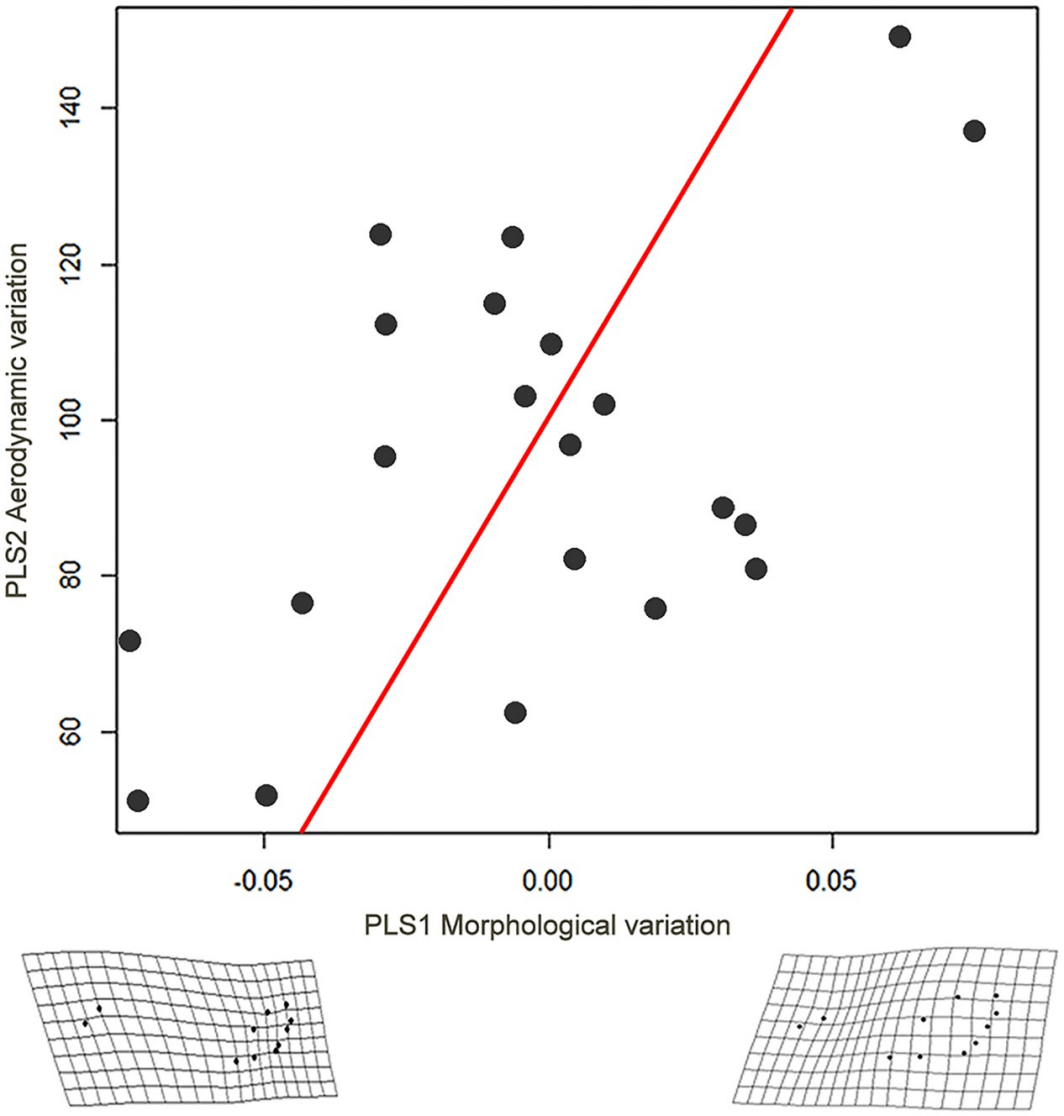
Partial Least Squares (PLS) regression, showing the relationship between wing shape and aerodynamic variables in females (2016 & 2017) of *H*. *charithonia*.

**Fig 6:**
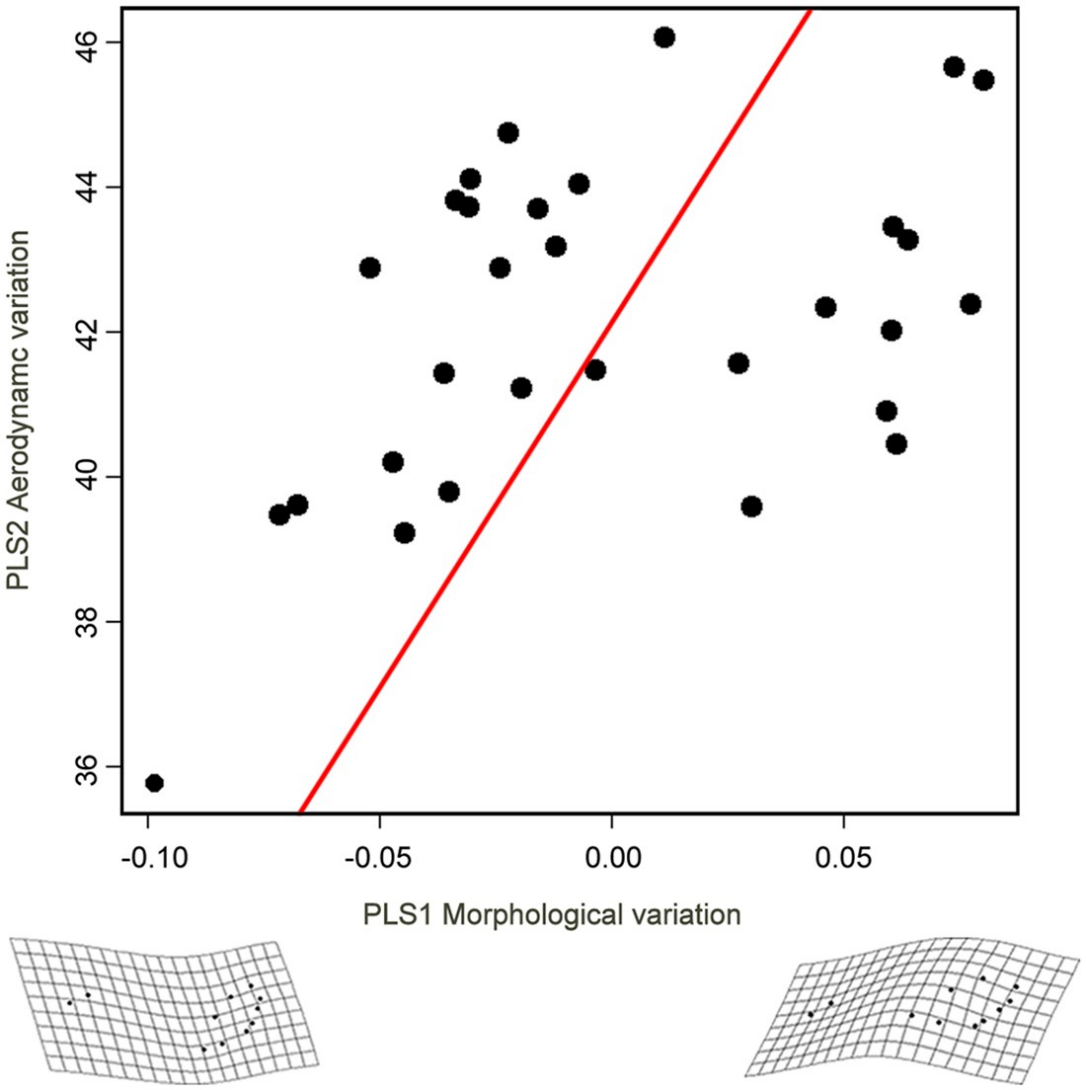
Partial Least Squares (PLS) regression, showing the relationship between wing shape and aerodynamic variables in males (2016 & 2017) of *H*. *charithonia*.

Female wing shape was significantly related to the aerodynamic variation (r = 0.5572, p = 0.0231, Table 4); the axis PLS1 of wing shape variation showed a shortening of the wings (Fig 5) correlated with the increased morphological variation (axis PLS2 aerodynamic variation), in which dry body mass and wing loading explained the majority of the covariation (Table 4). Also the wing shape tended to be related to variation in female energetics (r = 0.5289, p = 0.0614, Table 4). However, no relationship was observed between wing shape and the metabolic rates or the energetic variables (Table 4). Also, male wing shape was significantly related to the aerodynamic variation (r = 0.4454, p = 0.0449, Table 4), with increasing aerodynamic variation (axis PLS2), the male wing widens at the base, whereas distally the wing narrows (axis PLS1; Fig 6). However, for males, dry body mass, wing length and aspect ratio explained the majority of the covariation (Table 4). In males, no relationship was observed between wing shape and the metabolic rates or the energetic variables (Table 4).

**Table 4:** Results of the multiple Partial Least Squares (PLS) regressions of *H*. *charithonia*.

		Females N = 21		Males N = 30	
Aerodynamic variables		r-PLS	*P*-value	r-PLS	*P*-value
		0.5572	**0.0231**	0. 4454	**0.0449**
**Component**	Variable	**PLS 1**		**PLS 1**	
**1**	Dry body mass	0.7060		0.5176	
**2**	Wing length	0.4689		0.5158	
**3**	Wing loading	0.6942		0.3576	
**4**	Proportion of flight muscle	0.3806		0.3741	
**5**	Aspect ratio	0.3542		0. 4926	
**Metabolic variables**		r-PLS	*P*-value	r-PLS	*P*-value
		0. 2951	0.44	0. 4054	0.0879
**Component**	Variable	**PLS 1**		**PLS 1**	
**1**	Resting metabolic rate	0.5162		0. 3088	
**2**	Post-flight metabolic rate	0. 6317		0. 3631	
**3**	Post-flight metabolic rate (second minute average)	0. 6456		0. 3417	
**Energetic variables**		r-PLS	*P*-value	r-PLS	*P*-value
	PLS	0.5289	0.0614	0.2439	0.561
**Component**	Variable	**PLS 1**		**PLS 1**	
**1**	Carbohydrates Thorax	0.7225		0.4367	
**2**	Carbohydrates Abdomen	0.4386		0.1680	
**3**	Lipids Thorax	0.3761		0.3283	
**4**	Lipids Abdomen	0.272		0.3943	
**5**	Proteins Thorax	0.4295		0.2266	
**6**	Proteins Abdomen	0.4992		0.3308	
**7**	Total Carbohydrates	0.4636		0.3824	
**8**	Total Lipids	0.3256		0.2460	
**9**	Total Proteins	0.2454		0.2533	
**10**	Proportion of lipids in thoracic muscles	-0.1076		-0.0493	

The significant relationships are in bold.

## Discussion

Organisms that differ in their flight morphology can have different behavioral repertoires or differ in their performance during particular behaviors. Studies of the evolutionary ecology of flying insects, such as butterflies and dragonflies, have considered that differences in design (i.e. wing morphology) affect the adaptation of individuals, assuming differential performance. In the case of *H*. *charithonia*, it has been suggested that differences presented in flight morphology between the sexes, and between the males with different mating strategies, have implications for locomotor performance and for patterns of metabolic expenditure and resource allocation (i.e. energetic reserves) [39].

This study evaluated the relationship between wing morphological variation and metabolic performance of females and males of *H*. *charithonia* in the same population in which both inter and intrasexual differences in flight biomechanics have been documented previously [11]. The results confirm the occurrence of sexual dimorphism of wing shape and size in both years of the study. This could be associated, as suggested by Mendoza-Cuenca & Macías-Ordóñez [39], with differences between both sexes in flight requirements related to foraging preferences, egg production and searching behavior for mates. Our results coincide with those reported in many species of the genus *Heliconius* (including *H*. *charithonia*) in that wing shape can be molded by different pressures of sexual selection [13, 26, 27, 37–39, 57, 58]. When we compared both years, we observed contrasting intrasexual patterns; while females‘ wing shape did not differ between years, males presented marked inter-annual differences. Similar patterns have been described in other butterfly species [30, 49, 59–61], suggesting strong pressures of natural selection that restrict morphological variation in the females due to selective pressure derived from carrying eggs. While, in the case of the males, morphological plasticity seems to respond to selective pressures from both environmental challenges and mating tactics, maintaining an optimum flight design.

It was also found that females had greater wing length than males in both years, and that wing loading (WL) was significantly related to wing shape, probably because the additional mass of ovarioles requires increasing WL in order to enable displacement [10, 21], and also allow faster flight avoiding predation [62]. An interesting finding was the lack of seasonal or inter-annual variation in mean wing length in both sexes, similar also to average values reported by Mendoza-Cuenca & Macías-Ordóñez [11, 39], between 2000 and 2004. This contrasts with the seasonal and inter-annual plasticity in wing size observed in other butterfly species including *Heliconius*, related to seasonal [63–67] changes in larval quality, quantity and even host plant species of their larvae [30, 63–65]. In the absence of evidence of high heritability of this trait, the lack of intrasexual plasticity in wing size of males and females over time could reflect an absence of feeding restrictions for their larvae. At our study site, *H*. *charithonia* is the only species that oviposit in *Passiflora adenopoda* [39], a common species in the area, reaching large sizes and presenting a high and continuous availability of growth meristems. The solitary oviposition behavior of this species in which females oviposit one or few eggs in each shoot of *P*. *adenopoda* may guarantee enough resources for the development of their larvae [68]. *H*. *ismenius* and *H*. *hortense* are also present in our study site, have also maintained their average wing size between years (Ramos-Pérez, unpublished data).

Our results suggest the presence of considerable plasticity in wing shape in *H*. *charithonia*, possibly associated with optimization of morphology in the face of environmental effects on other phenotypic traits (i.e. wing length, body mass and length) that could improve speed, maneuverability and flight efficiency [62]. In this context, it has been suggested that aerodynamic traits such as wing loading and wing aspect ratio are closely related to the energy required for flight, and the availability of nutrients depends entirely on feeding efficiency. In a study conducted by Niitepõld *et al*. [23], which measured the effects of diet restriction on the metabolic rates of the butterflies *Speyeria mormonia* and *Colias eurytheme*, these authors found that food availability affects body mass, which in turn directly modifies wing loading but not flight capacity, and therefore suggested that the individuals are equally efficient in terms of obtaining sufficient energy to maintain flight capacity, or that they use metabolic strategies that limit the use of nutrients destined for other purposes, such as reducing their investment in reproduction or their daily activity. Contrary to our predictions and despite wing-shape differences, the results pertaining to resting metabolic rates do not show differences between males and females in *H*. *charithonia*, suggesting an optimization of metabolic performance in both sexes probably associated with access to a large number of essential amino acids and protein by feeding on pollen (as in many species of *Heliconius*) [32, 36]. Some of this protein subsequently participate in metabolization of carbohydrates [32, 36, 42], and may optimize the assignation of “fuel” for flight, maintaining a high metabolic capacity [69], allowing the individuals to meet the requirements imposed by the shape of their flight apparatus, the behaviors and daily patterns of flight, as well as the reproductive requirements of each sex (e.g. egg production vs searching for mates).

While we cannot rule out the possibility that individuals may be restricting their energetic expenditure on reproduction or flight activity and maintaining an optimum basal metabolism, previous evidence suggests that it is not the case in the genus *Heliconius*, since the females produce eggs at a constant daily rate throughout their lives without show any evidence of senescence [68]. In the case of the males in this same population, behavioral observations for three consecutive years showed that they fly daily in the reproduction areas in search of receptive females, and fly for more hours than the females [39, 43]. These observations together with our results suggest that male wing shape variation could allow them to optimize the energetic expenditure required for flight during foraging and mate seeking behaviors. Males consume mainly nectar or at least less pollen than females [11]; therefore, their source of carbohydrates is greater than that of the females and, given that carbohydrates are the main fuel that is oxidized metabolically and used for flight muscles, energy not spent in flight can then be stored.

In contrast with findings in other butterfly species in which the resting metabolic rate decreases with age (i.e. metabolic senescence) and with increased flight effort [13, 23–25], our results suggest that age does not affect the metabolic performance in either sex in *H*. *charithonia*. While we cannot assess the exact age of the individuals in which RMR and PFMR, the estimated age range is relatively wide (15–45 days, considering the marking and recapture period) and is not reflected in the obtained estimates of metabolic rates. This differs from that reported in other species using similar or even narrower age ranges, in which effects on both flight and peak metabolic rates have been reported [13, 23–25].

In general, our results confirm the expected intersexual differences in the biomechanical design of flight in *H*. *charithonia*, but also suggest that pressures of natural and sexual selection could be involved in optimizing wing shape, increasing aerodynamic efficiency or reducing energy losses in flight, which would explain the similarities observed in metabolic rates and energy reserve between females and males, and even between individuals of different ages.

## Supporting information

S1 Table Aerodynamic variables measured in individuals of *H*. *charithonia* during 2016 and 2017 (mean ± SD). Different letters show significant differences according to the Tukey-Kramer test. Significant differences in the two-way ANOVA are in bold.
(DOC)

S2 Table Variables measured in individuals of *H*. *charithonia* during 2016 and 2017 (mean ± SD). (DOC)
