# Supplementary material for: Sex-related interannual plasticity in wing morphological design in Heliconius charithonia enhances flight metabolic performance
Source: PLoS One. 2020 Oct 30;15(10):e0239620. doi: 10.1371/journal.pone.0239620 (PMC7598497; doi:10.1371/journal.pone.0239620)
Supplement: S1 Table — Different letters show significant differences according to the Tukey-Kramer test. Significant differences in the two-way ANOVA are in bold. (DOC) [file pone.0239620.s001.doc]

**S1 Table. Aerodynamic variables measured in individuals of *H. charithonia* during 2016 and 2017 (mean ± SD).** Different letters show significant differences according to the Tukey-Kramer test. Significant differences in the two-way ANOVA are in bold.

|  | **Females 2016** | **Females 2017** | **Males 2016** | **Males 2017** | **F** | **p** | **d.f.** |
| --- | --- | --- | --- | --- | --- | --- | --- |
|  | **N=18** | **N=37** | **N=35** | **N=39** |  |  |  |
| **Wing length (mm)** | 44.97±2.87a | 44.72±3.13a | 41.44±2.43b | 41.84±2.77b | 11.98 | **<0.0001** | 3 |
| **Body length (mm)** | 24.83±1.63ab | 25.44±1.80a | 24.29±1.53b | 24.82±1.78ab | 2.73 | **0.0454** | 3 |
|  | **N=7** | **N=14** | **N=11** | **N=19** |  |  |  |
| **Dry body mass (g)** | 0.092±0.03a | 0.089±0.02a | 0.067±0.01a | 0.080±0.02a | 2.74 | 0.053 | 3 |
| **Dry thoracic mass (g)** | 0.042±0.01a | 0.043±0.01a | 0.032±0.01a | 0.037±0.01a | 2.04 | 0.12 | 3 |
| **Dry abdominal mass (g)** | 0.031±0.01ab | 0.031±0.01a | 0.019±0.003c | 0.023±0.01bc | 6.02 | **0.0015** | 3 |
| **Wing loading (Nm^-2^)** | 0.51±0.1a | 0.47±0.07a | 0.42±0.07a | 0.49±0.08a | 1.91 | 0.14 | 3 |
| **Proportion of flight muscles** | 0.45±0.05a | 0.48±.05a | 0.46±.07a | 0.47±.05a | 0.68 | 0.56 | 3 |
| **Aspect ratio** | 4.48±0.22a | 4.42±0.20a | 4.59±0.17a | 4.42±0.18a | 1.67 | 0.18 | 3 |
