# Supplementary material for: Sex-related interannual plasticity in wing morphological design in Heliconius charithonia enhances flight metabolic performance
Source: PLoS One. 2020 Oct 30;15(10):e0239620. doi: 10.1371/journal.pone.0239620 (PMC7598497; doi:10.1371/journal.pone.0239620)
Supplement: S2 Table — (DOC) [file pone.0239620.s002.doc]

**S2 Table. Variables measured in individuals of *H. charithonia* during 2016 and 2017 (mean ± SD).**

|  | **Females 2016**  **N=7** | **Females 2017**  **N=14** | **Males** **2016**  **N=11** | **Males 2017**  **N=19** |
| --- | --- | --- | --- | --- |
| **METABOLISM** |  |  |  |  |
| **Resting Metabolic Rate**  **(ml CO_2_ h^-1^)** | 0.14±0.0.06 | 0.14±0.09 | 0.10±0.03 | 0.12±0.09 |
| **Post Flight Metabolic Rate**  **(ml CO_2_ h^-1^)** | 1.39±0.91 | 1.46±0.88 | 1.59±0.91 | 1.54±1.07 |
| **Post Flight Metabolic Rate maximum (ml CO_2_ h^-1^)** | 0.46±0.38 | 0.37±0.25 | 0.56±0.31 | 0.34±0.26 |
|  |  |  |  |  |
| **ENERGETICS** |  |  |  |  |
| **Total carbohydrates (µg/mg)** | 440.21±331.52 | 365.89±291.57 | 194.89±137.51 | 200.52±147.54 |
| **Total lipids (µg/mg)** | 375.83±275.78 | 370.75±260.32 | 177.56±123.74 | 263.06±226.24 |
| **Total proteins (µg/mg)** | 45.92±31.07 | 35.33±21.67 | 24.37±11.49 | 22.91±18.32 |
| **Proportion of lipids present in the thoracic muscles (mg)** | 0.5279±0.06 | 0.4880±0.06 | 0.5427±0.09 | 0.4746±0.05 |
